# Applications of Deep Learning for Dense Scenes Analysis in Agriculture: A Review

**DOI:** 10.3390/s20051520

**Published:** 2020-03-10

**Authors:** Qian Zhang, Yeqi Liu, Chuanyang Gong, Yingyi Chen, Huihui Yu

**Affiliations:** 1 National Innovation Center for Digital Fishery, China Agricultural University, Beijing 100083, China; SY20183081452@cau.edu.cn (Q.Z.); liuyeqi@cau.edu.cn (Y.L.); sy20173081267@cau.edu.cn (C.G.); 2 College of Information and Electrical Engineering, China Agricultural University, Beijing 100083, China; 3 Beijing Engineering and Technology Research Center for Internet of Things in Agriculture, Beijing 100083, China; 4 Key Laboratory of Agricultural Information Acquisition Technology, Ministry of Agriculture, Beijing 100083, China; 5 School of Information Science & Technology, Beijing Forestry University, Beijing 100083, China; yuhh1990@bjfu.edu.cn

**Keywords:** deep learning, dense scenes, agricultural application, computer vision

## Abstract

Deep Learning (DL) is the state-of-the-art machine learning technology, which shows superior performance in computer vision, bioinformatics, natural language processing, and other areas. Especially as a modern image processing technology, DL has been successfully applied in various tasks, such as object detection, semantic segmentation, and scene analysis. However, with the increase of dense scenes in reality, due to severe occlusions, and small size of objects, the analysis of dense scenes becomes particularly challenging. To overcome these problems, DL recently has been increasingly applied to dense scenes and has begun to be used in dense agricultural scenes. The purpose of this review is to explore the applications of DL for dense scenes analysis in agriculture. In order to better elaborate the topic, we first describe the types of dense scenes in agriculture, as well as the challenges. Next, we introduce various popular deep neural networks used in these dense scenes. Then, the applications of these structures in various agricultural tasks are comprehensively introduced in this review, including recognition and classification, detection, counting and yield estimation. Finally, the surveyed DL applications, limitations and the future work for analysis of dense images in agriculture are summarized.

## 1. Introduction

Nowadays, smart agriculture [1] has become increasingly popular because it can adjust various agricultural management measures accurately according to the specific conditions of each unit of agricultural operation. In order to optimize the input of agricultural production links, almost all the management models in agriculture are based on the models of high-density planting and mass production, thus achieving the goal of obtaining the maximum economic and environmental benefits. However, in actual production and life, it is inevitable to produce a lot of dense images. It is worth taking note that the complexity of the problem increases exponentially with the number of objects and the interaction of the objects. Especially, the pattern of batch production and high-density planting determines the high density of agricultural scenes, and high density makes it difficult to meet the actual demand, such as detection, counting or yield estimation of agricultural products.

In recent years, due to the tremendous progress and popularity of image acquisition equipment, the image has become a huge amount of data and easy to obtain, which makes image analysis very meaningful and challenging [2]. With the significant reduction of computer hardware cost and the significant improvement of GPU computing power, the computer vision task has become an important area of machine learning and artificial intelligence. In general, machine learning combined with high-performance computing guarantees the ability to process large amounts of image data effectively.

Early solutions to computer vision tasks depended on traditional machine learning methods, i.e., feature-based manual method. Common features include Deformable Part-Based Model (DPM) [3], Histogram of Oriented Gradient (HOG) [4] and Scales-Invariant Feature Transformation (SIFT) [5], Speeded Up Robust Features (SURF) [6] and Haar-like features [7]. They were usually combined with classifiers such as Support Vector Machine (SVM) to classify each image pixel. Although the traditional methods are easy to understand and many improvements have been done to them, most of them are verified in low and medium density images and they usually need to be changed according to the specific situations [8,9]. Moreover, most traditional methods either ignore the problems in dense scenes, namely, there is no discussion on dense scenes, or use simple heuristic methods based on shape and size, these methods are very ineffective in natural environments with severe occlusion and large scale changes [10]. Therefore, traditional machine learning methods are not appropriate for dense images. It has been demonstrated that in many applications, features extracted by deep learning are more effective than these hand-crafted feature [11]. Moreover, deep learning solves various challenges in dense images.

Deep Learning (DL) [12] belongs to machine learning, based on representation learning of data, which realizes artificial intelligence by means of artificial neural networks with many hidden layers and massive training data. DL has been successful in computer vision [13], natural language processing [14], bioinformatics [15], automatic control [16], machine translation [17], automatic driving [18] and other practical problems [19]. The reason for the success of DL lies in its unique characteristics of network structure: deep neural network can acquire high-level features by learning local features from the bottom and then synthesizing these features at the top. DL uses multi-level abstraction to learn complex feature representations from raw data and generate components automatically [20]. Different features at different levels can correspond to different tasks. Deep learning is a technology that uses deep network structure to learn features. Deep learning emphasizes the depth of the model structure, highlights the importance of feature learning and proposes various techniques to learn more and higher-level features better and faster. Strong learning ability enables them to implement various kinds of problems especially well and flexibly adapt to numerous highly complex problems. Monitoring and studying a large number of interesting objects in videos or images is an important task for the macro-world and micro-field, for instance, the research on crowding traffic and microscopic microorganisms [21,22].

Usually, advances in one area are driven more by some combination of expertise, resources, and application requirements in other areas [23]. Similarly, applications of DL in analyzing dense scenes spread to the agricultural sector after advances in medical diagnostics and population analysis. More and more scenes in agriculture produce a lot of high-density images, and they are becoming more and more attention. At present, agricultural tasks have been transformed into tasks of agrovision (computer vision in agriculture). There have been some reviews on the applications of DL in agriculture [24,25] and some reviews pertinent to the use of DL in computer vision. They either gave a comprehensive overview of DL methods applied throughout the agricultural field or the latest research of DL technology in a certain agricultural field and also reviewed the application of DL methods in general computer vision tasks. However, none of them involved how DL works in dense agricultural scenes. Koirala et al. [23] summarized the use of DL in fruit detection and yield estimation, including the problem of occluded fruit in imaging and the solutions. However, they were only concerned with the detection and yield estimates while ignoring other agricultural tasks containing a large number of objects. Thus, the motivation for preparing this review stems from the need to summarize the applications of DL in agriculture with the increase of dense scenes and images.

### 1.1. Types of Dense Scenes in Agriculture

Dense scenes mentioned in most papers refer to the videos or images about the crowds, and a lot of studies have been done in the field of automatic monitoring and crowd analysis [26,27]. To the best of our knowledge, No one seems to define dense scenes in agriculture so far. In this section, by observing various scenes in agriculture, we distinguish whether the scene is dense or not according to the number of interesting objects with specific tasks, namely, there are two conditions to judge whether a scene is dense. One is that there are a large number of interested objects in the image. The other is the case that the research task is to detect/classify each object. Based on these available options, two types of dense scenes in agriculture were formulated:

(1) Quantity-dense scenes

**Figure 1:**
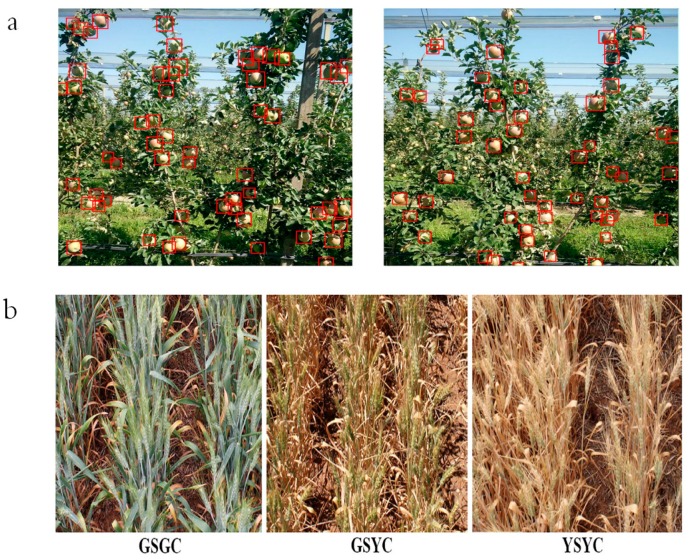
Dense scenes in agriculture. (**a**) Detecting fruits in an orchard. Source: [31] is licensed under https://creativecommons.org/licenses/by/4.0/; (**b**) Green Spike and Green Canopy (GSGC), Green Spike and Yellow Canopy (GSYC), Yellow Spike and Yellow Canopy (YSYC). Source: [32] is licensed under https://creativecommons.org/licenses/by/4.0/.

Quantity-dense scenes (Figure 1a) refer to images or videos with a very large quantity of objects, which are often accompanied by severe occlusion and overlap. Although many systems can assess agricultural crops under controlled conditions, they ignore the interaction between crops and the environment, and many crops have to be evaluated directly in their growth environment. For example, fruit in orchards, grain in fields, vegetation in grasslands, etc. While in practice, producers usually adopt the way of high-density planting for agricultural production. In actual orchards, fruit is densely distributed and usually overlapped by each other, the ubiquitous branches and leaves even shade the fruit. Grain fields usually have very narrow row spacing when planting high-density crops, and overlapping plants often appears.

However, it should be noted that the images acquired in the above scenes are not necessarily what we call dense scenes. We should judge the scenes according to the specific tasks, such as in [28], which is also to study farmland, but the task is to classify different farmland types, and it does not care whether the grain distribution in farmland is dense or not, so we do not divide such scenes into dense scenes. The problems in analyzing this type of dense scenes are usually the same and the solutions are similar.

(2) Inner-dense scenes

The second type of dense scenes refers to some objects in an image which have dense attributes themselves, i.e., the objects in the images are the same kind of objects that are conglutinated with each other (Figure 1b), such as wheat, grapes, hives, etc. The study of these objects, especially a wheat spike or an ear of rice, is often a study of dense objects. In this case, separating adjacent objects is the biggest challenge. That is to say, because of the large number and the interaction between adjacent objects, data labeling is a huge problem, not to mention the use of DL methods for subsequent processing. Due to the nature of those objects, these phenomena exist in every image of them. Therefore, the research on the second type of dense scenes is less than that of the first type.

With the deep study of agricultural production, it has been recognized that most scenes in agriculture are dense or crowded scenes. Therefore, analysis of agricultural problems mostly needs to analyze the dense scenes. As shown in Figure 1, the high-density objects in specific agricultural tasks pose severe challenges to the analysis of agricultural dense scenes, especially for underwater fish or other aquatic objects [29,30]. Consequently, more and more attention should be paid to the research of dense scenes in agriculture.

### 1.2. Challenges in Dense Agricultural Scenes

**Figure 2:**
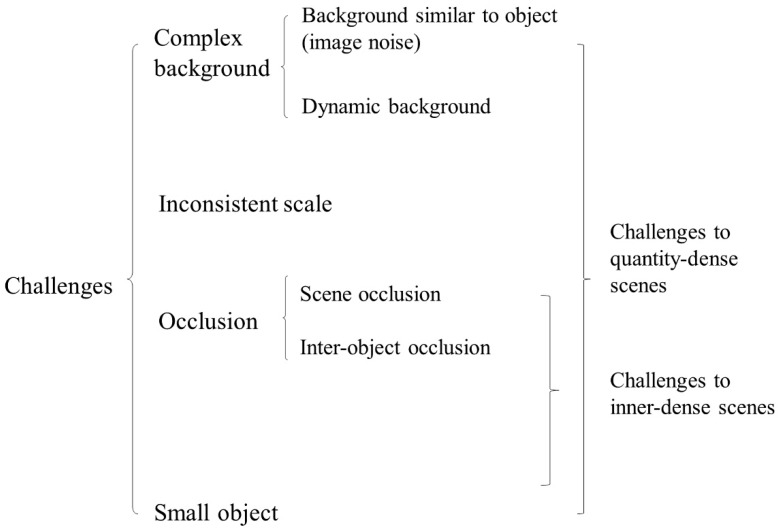
Challenges in dense scenes in agriculture.

Research has found that there are a variety of thorny problems in dense scenes, as illustrated in Figure 2. First of all, there are some challenges in both quantity-dense and inner-dense scenes in agriculture. Sa et al. [33] pointed out that occlusion is a key challenge in dense scenes. There are two types of occlusion: scene occlusion and inter-object occlusion. Scene occlusion refers to the occlusion of the detected object by other non-objects; inter-object occlusion means that the detected objects in the image are occluded from each other, which is at the heart of the occlusion problem. It should be noted that in the quantity-dense scenes, there are two kinds of occlusion. However, for the inner-dense scenes, there is often no occlusion of other objects in the image, so the main challenge is inter-class occlusion. In addition, high-density objects will lead to a small size of a single object and the object resolution is relatively low in both types of agricultural dense scenes. Moreover, because of the small size of the object, it is easily affected by noise and leads to inaccurate detection, and there is a general limitation in agricultural applications, namely, sensitivity to changing illumination conditions. Although it has been proved that LIDAR sensors are reliable for solving this problem, they usually require higher investment costs. Besides, objects at different distances from the camera may vary greatly in scale, bringing about constant changes in imaging features, especially in object size.

Meanwhile, there are some unique challenges for the quantity-dense scenes in agriculture, i.e., complex background. The complex background includes two situations, one is that the background is similar to the object, and the noise is intense, which makes it difficult to distinguish the object from the background. The other is the case that the background may be dynamic, which changes with the moving of the object. Images obtained from realistic agricultural scenes such as farmland or orchard tend to have high background noise, due to the nature of this challenge which is an unpredictable event, the methods are so varied and there is no robust solution that works in all conditions.

These challenges may occur in other computer vision tasks, but more frequently or simultaneously in dense scenes. Thus, methods specifically for dense scenes in agriculture need to be proposed.

### 1.3. Outline

As far as we know, this is the first such overview of the applications of DL in dense scenes of agriculture. We summarize various DL algorithms used in dense scenes, which will contribute to the application of DL algorithms in a natural agricultural environment. The rest of the paper is organized as following:

In Section 2, two categories of DL models, the most widely used models in dense scenes are reviewed: Convolutional Neural Networks (CNN) and Autoencoder, as well as the basic architectures, contributions, and limitations of each category. Then we introduce the achievements of those DL algorithms above to various agriculture applications in Section 3, including recognition, classification, detection, counting and yield estimation. Emphasis is placed on how the DL model handles dense scenes. Finally, we terminate this paper with a discussion and a conclusion. We hope this review can provide new ideas and suggestions researchers who are engaged in dense scenes analysis in agriculture and be satisfactorily used to develop expert or intelligent systems in other related fields.

## 2. Deep Learning Methods

**Figure 3:**
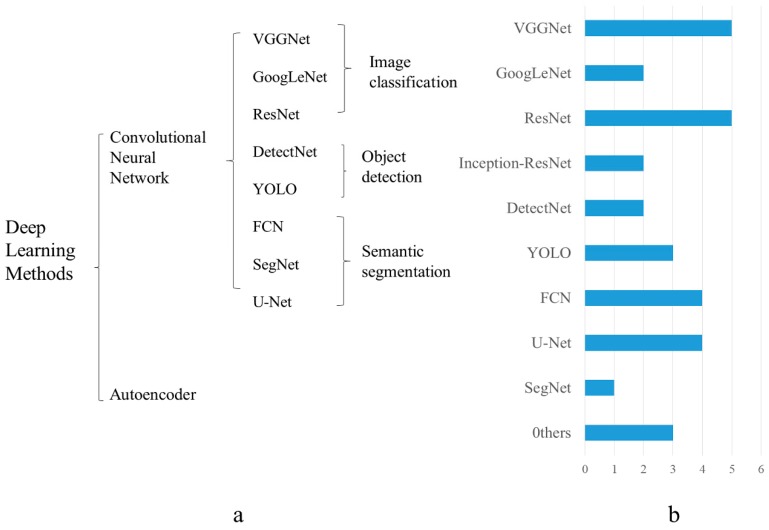
(**a**) Different architecture of Deep Learning (DL) Algorithms in dense agricultural scenes; (**b**) Uses of surveyed DL methods.

**Figure 4:**
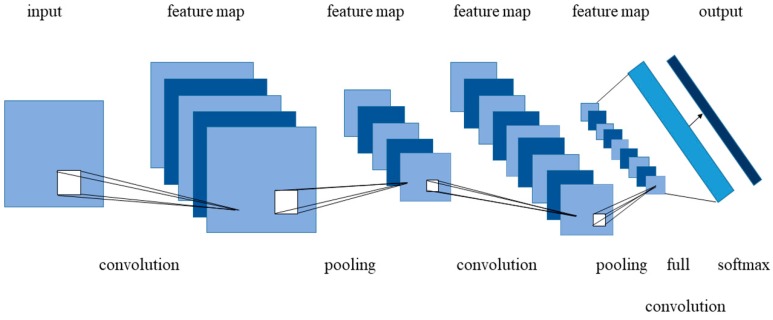
Overall architecture of Convolution Neural Networks.

DL has shown an incomparable advantage for computer vision tasks in the context of big data. Especially in the tasks of detection, classification, and identification, DL technology occupies a core position at present. However, these methods are difficult to apply directly to dense scenes. Therefore, in this section, we focus on two DL methods that work well in dense agricultural scenes and introduce the popular backbone networks among them. As illustrated in Figure 3, two DL methods are respectively: Convolutional Neural Networks (CNN) and Autoencoder. Table 1 displays the characteristics of various backbone networks and their highlights in dense scenes. There are many other deep learning structures that have been applied in agriculture, but have not been used in agricultural dense scenes. For example, Recurrent Neural Network (RNN) and Long Short-Term Memory (LSTM) are very useful in processing time-series data, which are typically used in agriculture for time prediction. Generative Adversarial Network (GAN) can be used to enrich datasets and it has been applied in agriculture [34,35], but it has not been widely used in agricultural dense scenes. In addition, Figure 4 shows the uses of surveyed DL methods in a recent study of dense scenes in agriculture. This chart information is very valuable to new researchers since it can help them find the most effective DL algorithm and further research.

### 2.1. Convolutional Neural Networks (CNNs)

#### 2.1.1. Review of CNNs

CNN is a feedforward neural network proposed by Fukushima [41] for the first time, the essence of which is a Multi-Layer Perceptron (MLP). Despite some modifications, the core concept of a neural network has not changed much. It consists of the input layer, hidden layers, and output layer, each layer has hundreds of nodes. The nodes in the previous layer are connected to other nodes in the next layer by weight, which reflects the strength of the connection between the two nodes. Figure 4 shows the overall architecture of the CNNs: as a whole, the CNNs can be divided into three main types of neural layers, namely, convolutional layer, pooling layer, and fully connected layer. Various CNNs have different implementation details. The function of the convolutional layer is to extract features from input data, which contains multiple kernels. Because the spatial connection of the image is local, each neuron only needs to feel the local image area, and CNN can get the global information at a higher level. The values in the convolution kernel is the weights, and the weights of each local area in the same convolution layer are shared. There are many initialization methods for weight, such as random initialization, pre-training, etc., and the weights are updated during model training. Using different kernels, various feature maps can be generated. The reason for the success of convolutional layer and popularity to date lies in the way of its local connectivity and weight sharing [42], which reduce the number of weights to reduce the complexity of the model and the risk of over-fitting, as well as making the network easy to optimize. The pooling layer, also known as the sub-sampling layer or down-sampling layer, is often behind the convolutional layer, which is the operation of feature selection and filtering for the input feature map. On the one hand, it makes the feature map smaller and simplifies the complexity of network computing; on the other hand, it compresses the feature, extracts the main features, and ensures the invariance of feature position and rotation. Average pooling and max pooling are the most commonly used strategies, among which max pooling is used more because of its better performance [43]. The fully connected layer is usually the last part of the hidden layer of CNNs. Its function is to integrate the highly abstract features which have been convoluted and pooled many times before, that is, to connect all the features into one vector, then, the expanded vector is passed to the classifier through the activation function for classification or further processing [44]. Research has shown that the representation learned by CNN is rich in an amazing amount of image information, including position, size and pose of objects [45]. The training process of CNNs can be divided into two stages. The first stage is called forward propagation stage. Data flows from the input to the output, and the network extracts the features from the low level to the high level. Another stage is called the back-propagation stage, which aims to adjust the weight and bias to minimize the overall cost (error). Follow-up works improved CNNs to enable it to be more effectively learn and extract the features, which have great advantages in the process of 2-D image processing. Now, 3D convolution characterized by 3D convolution kernels for the processing of videos is commonly used [46]. In the analysis of dense scenes, most of the research works use CNNs as the framework.

#### 2.1.2. CNNs’ Backbone Network

There are many popular and advanced backbone networks of CNNs. Although most deep CNNs are composed of a set of base layers introduced above, each network has its characteristics and suitable application scenes; not all networks are suitable for dense scenes. Therefore, in this section, we will focus on the backbone networks which are most used and work well in dense scenes. Including classification networks: VGGNet, GoogLeNet, ResNet, detection networks: DetectNet, YOLO, and semantic segmentation networks: FCN, SegNet and U-Net. Most of the works reviewed in this paper are based on these network structures for combination or fine-tuning, making it more suitable for analyzing dense scenes in agriculture. 

Visual Geometry Group Network (VGGNet) [36] explores the relationship between the depth and performance of a convolution neural network. VGGNet inherits some frameworks of LeNet [47] and AlexNet [44]. However VGGNet uses the small convolution filter size. The outstanding contribution of VGGNet is to prove that using a very small convolution filter size and increasing the depth of the network can effectively improve the performance of the model. At the same time, it has strong expansibility, and good generalization ability for other datasets makes it a valuable network for transfer learning.

Different from previous structures of AlexNet and VGGNet, which increase the depth of the network to improve training results, GoogLeNet [13] is a novel DL structure. To build a sparse and high computing performance network structure, an inception module is proposed in the GoogLeNet inspired by that sparse matrices can be clustered into more dense sub-matrices to improve computing performance. Convolution and reassembly of multiple sizes can extract the features of different scales and accurately gather the features with a strong correlation in advance, thus accelerating the convergence of the network. DetectNet is a GoogLeNet-based network for optimizing object detection. It consists of two parts: fully convolution neural network and clustering function. The fully convolution neural network is based on the GoogLeNet structure without the input layer, the final pool layer, and the internal product layer. Allowing the use of pre-trained GoogLeNet to initialize DetectNet reduces training time and improving the accuracy of the final model. DetectNet not only predicts the existence of the object but also predicts the position of the boundary angle of the object relative to the center of the grid square, this is the main advantage of using DetectNet for object detection.

The original inspiration of Residual Network (ResNet) [37] is a problem called degradation, that is, accuracy will rise first and then reach saturation, and continuous increase in depth of the net will lead to a decline inaccuracy. As the convergence of deep network and accuracy reaches saturation, the network begins to degenerate. This problem is solved in ResNet by a structure consisting of several layers of networks containing a shortcut connection called a residual block. Mask R-CNN [48] uses ResNet and Feature Pyramid Networks (FPN) [49] to fuse and extract multi-layer features. Shortly after ResNet was launched, Google borrowed the essence of ResNet and proposed Inception-v4 [50] and inception-residual-v1/v2 [51]. Inception-residual network combines the inception idea, which links the results of convolutional layers with different filter sizes through inception module to capture the characteristics of multiple sizes.

The two-stage method represented by R-CNN has higher and higher detection accuracy in computer vision tasks, but it is sometimes difficult to meet the real-time requirements in some scenes. One-stage method represented by YOLO [52] integrates feature extraction, candidate frame regression, and classification into a network, which improves the detection speed. YOLO is improved based on the GoogLeNet architecture, and the difference is that YOlO uses 1 × 1 + 3 × 3 convolution kernels to replace the inception module. Nevertheless, it has a problem in detecting small objects since the YOLO model can only detect one object class in each cell. So, when using this model in dense images, different changes are usually made to the initial model. Later, YOLOv2 [53] and YOLOv3 [54] are therefore proposed to improve it. In addition, new classification models, DarkNet-19 and DarkNet-53, are proposed in YOLOv2 and YOLOv3. Using ResNet for reference, both of them are mainly composed of a series of convolution kernels of 1 × 1 and 3 × 3 to avoid gradient dispersion and explosion of depth network. 

Fully Convolutional Networks(FCN) [38] is a kind of network that uses the convolutional layer instead of the fully connected layer in the previous classification network. In dealing with dense images, the segmentation of objects is the basic work of many tasks. FCN is one of the first successful semantically segmentation method [55,56]. There are three main technologies used in FCN: convolutional, up-sample and skip layer. The existence of a fully connected layer will lead to a large storage overhead and constrain local features. Then FCN transforms the last three layers of CNN into three layers of the convolutional layer, which is used for end-to-end and point-to-point semantic segmentation. 

As an effective image segmentation method, FCN has some limitations. For example, the blurring of the underlying features makes the network insensitive to the details of the image, and often ignores the relationship between the local and the whole. In addition, the pooling layer of CNNs discards part of the location information, semantic segmentation needs to retain the location information discarded in the pooling layer. As a result, networks based on encoder-decoder are proposed to solve this problem. SegNet [39] and U-Net [40] are the most prevalent semantic segmentation networks based on encoder-decoder, which are also used widely in dense scenes.

SegNet is modified by the VGG-16 network based on FCN. The encoder used in SegNet the first 13 layers of the convolution network of VGG-16. Each encoder layer corresponds to a decoder layer. In the encoder process and decoder process, the convolution method utilized by SegNet is the same convolution. The difference is that in the decoder process, convolution is used to make information lost in the pooling process available through the decoder.

U-Net is an improvement on FCN, consisting of a contraction path and an expansion path. The contraction path is used to get context information, and an expansion path is used to locate accurately. Different from FCN, U-Net uses skip connection to cascade the feature information of contraction path of the model with that of deconvolution operation in expansion path, so as to obtain multi-scale feature information to improve network performance. In order to recover the structural features of the original image, U-Net uses 4 skip connection to connect the low-level and high-level feature maps.

### 2.2. Autoencoder

#### Overview

Autoencoder network is a type of unsupervised learning method. The main objective of autoencoder is to automatically learn and represent the unmarked input data, typically for data dimensionality reduction, compression, fusion. Autoencoder includes two processes: encode and decode. Input images are processed by encoding to get code, and then decode to get output, by using the back-propagation algorithm to train the network so that the output equals the input. High-dimensional data is always difficult to understand; if we reduce the input data to a lower dimension, it will be much more intuitive and easy to handle.

**Figure 5:**
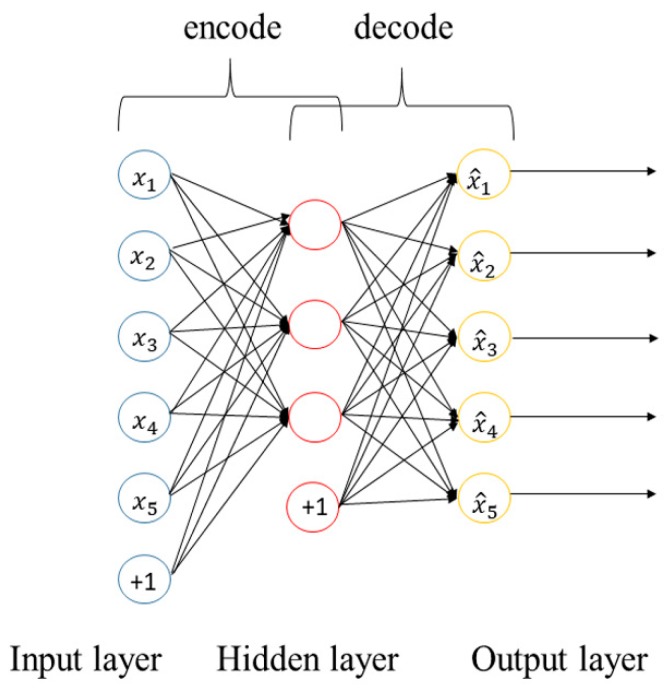
Conceptual diagram of autoencoder.

The following Figure 5 shows the conceptual diagram of an autoencoder. It is a kind of neural network aiming at reconstructing input information and it can give a better description of features than the original data. That is to say, there is the following relationship between the output layer and the input layer of the autoencoder network: $x_{i}\approx {\hat{x}}_{i}$ [57].

Generally, different variations of the autoencoder have been proposed to ensure robust features representation for machine learning applications. These include parse autoencoder [58], denoising autoencoder [59], stacked autoencoder [60], and contractive autoencoder [61].

### 2.3. Conclusion

These deep learning networks are used to solve various challenges in agricultural intensive scenes due to their own characteristics. To deal with the complex problems encountered in dense scenes, it is inevitable to deepen the depth of the network. Therefore, VGGNet has a broad application in dense scenes analysis. Convolution and reassembly of multiple sizes in GoogLeNet is an effective way to solve the problem of scale inconsistency in dense scenes. The training data of DetectNet is a large image containing multiple objects, which is suitable for dense object detection. The main advantage of ResNet is to destroy the flow of information in the network, but this means that some features can be reused, and the ResNet architecture is easier to optimize than other deep networks. Inception-residual network combines the inception idea, which links the results of convolutional layers with different filter sizes through inception module to capture the characteristics of multiple sizes. In addition, YOLO can recognize the background correctly, and can be used to detect a certain degree of occlusion and overlapping objects. The structure of FCN reduces the repeated calculation, reduces the complexity of the model with filling up the missing detail data during upsampling, and the input image can be any size. It is used for accurate crowd count estimation in crowded scenes [62]. As a semantic segmentation network based on encoder decoder, while the segmentation result of SegNet is not good, so in the dense agricultural image, U-Net is more used. The outstanding advantage of U-Net is that it can get very accurate segmentation results with very few training images. However, it still considers more high-level features and makes little use of low-level features. In general, analysis of agricultural dense scenes is one of computer vision tasks, and CNNs are the main deep learning method. At present, an autoencoder is rarely used in agricultural scenes.

## 3. Applications

Deep learning has been widely used in agriculture, such as disease diagnosis [62,63], crop recognition and classification [64,65], leaf counting [66], etc. To a great extent, these methods have solved the agricultural tasks. In fact, they cannot be applied directly to dense scenes. In most agricultural scenes, due to the existence of dense objects, these methods of DL cannot work well. Therefore, this section introduces the application of DL in dense scenes in agriculture. Table 2 shows the applications of deep learning networks in dense images in different agricultural tasks. We introduce the models used in various applications of agricultural dense scenes and the results. Among them, computing time indicates whether it is real-time. We found that in the dense agricultural scenes, the vast majority of datasets are created by the author. So, in Table 3, we describe the datasets used by the methods in Table 2.

### 3.1. Recognition and Classification

Object recognition is a process in which a particular object or a type of object is distinguished from other objects or other types of objects. Classification is the further processing of data based on recognition, that is, to recognize multiple classes. Recognition of agricultural dense scenes is mainly used in the recognition of pests and diseases and the recognition of objects as the basis of other tasks, such as fruit picking and object tracking. Classification tasks in dense scenes mainly include crop and weed classification and fine-grained classification in agriculture. In general, the recognition ability of the model increases as the depth deepens [36]. However, it has been shown that simply increasing the depth can cause network degradation [37]. Especially in dense scenes, it still faces the problem of complex background. Therefore, it is necessary to find a DL method to increase the depth of the model while restraining the degradation for dense scenes analysis in agriculture. Images in recognition tasks are quantity-dense scenes, while the images in classification tasks tend to be inner-dense scenes. Therefore, the problem of classification in dense scenes in agriculture is more complex. Usually, multiple DL methods are combined to deal with the overlapping and classification of different classes, i.e., classification-based network and semantic segmentation based network.

The main task of recognition in agriculture is to recognize diseases and insect pests. Timely and accurate diagnosis of plant diseases is one of the main objectives of precision agriculture [83]. Cheng et al. [63] used the 50-layer and 101-layer fine-tuning deep residual learning pre-trained on ImageNet to identify pests. Because of the end-to-end training on the pest dataset with a complex background, their system has strong robustness. The recognition accuracy of 98.67% for 10 classes of crop pest images with complex farmland background was achieved. Besides, recognition is the first step in picking in the orchard. The general system can only recognize the fruit, but in the field environment, it is also very important to recognize obstacles such as leaves and branches. Liu et al. [64] designed a fruit-picking robot working in a natural environment. Yolov3 and two Mark R-CNNs with backbone networks ResNet50 and ResNet152 were used. ResNet50 and ResNet152 contain 16 and 50 residual modules, respectively. The experimental results showed that the Mark R-CNN152 model has the highest comprehensive recognition accuracy of 85.12% and can meet the real-time requirements. To solve a series of problems such as random growth posture of trees and complex occlusion of branches and leaves to fruits, they divided the types of recognition into Normal Branch, Occlusion, Leaf Occlusion, Slight Occlusion, Overlapping and Main Branch according to the occlusion rate. Then they recognize, classify and plan the path according to the occlusion. Accurate individual recognition is an important step towards automated dense object tracking. In the hive, there are hundreds of dense, often occluded and constantly moving individuals. In order to identify all honeybee individuals in the dense natural environment, and judge the orientation of the individuals, Bozek et al. [65] used the method of combining U-Net and a recurrent component to encode the additional information of the segmented object. In their work, they reduced the size of U-Net to reduce overfitting. The proposed method can effectively identify individuals in iteration.

Crop and weeds are similar in shape, size, and color, so the classification of crop and weeds faces the challenges: the overlap between crop and weeds [84]. For general classification tasks, most of the methods use the fully connected layer of CNNs architecture for final classification. However, in dense scenes, the use of a fully connected layer directly for classification is not effective. Because the pooling layer compresses the original two-dimensional matrix into one-dimensional, thus, spatial information is lost. Therefore, classification tasks are usually divided into two steps in dense scenes. Firstly, object and background are segmented by semantic segmentation methods, and then foreground objects are classified. A new method combining robust pixel-wise with a coarse-to-fine classifier based on CNN was proposed in [66]. Specifically, the segmentation network was based on a modified version of U-Net architecture. The segmented network was trained by datasets from different contexts, plant types, fields and environmental conditions, which was used to generate the binary mask. Then the vegetation clumps in the binary mask were extracted and sent to the fine-tuned CNN classifier based on VGG-16 for crop/weed classification. The proposed method achieved good classification results on challenging data, i.e., serious occlusion of weeds and crops. It is the overlapping of weeds and crops that affects the detection results. Besides traditional fruit or crop classification, some tasks can also be transformed into classification tasks in analyzing dense scenes in agriculture, such as quality assessment, fine-grained classification and so on. In the study of [85], the evaluation of paddy field quality was transformed into the classification of paddy field density, including sparse density and normal density. Different from traditional disease classification, there are larger intra-class similarities and smaller inter-class variance [86]. So fine-grained classification in dense scenes is more of a challenge. At present, the research in this field is too little and not mature.

### 3.2. Detection

Detection is based on classification, which requires the network to output not only the category of the object but also the location parameters of the object. Accurate detection is usually the basis of other tasks. In agricultural applications, weed control, fruit picking and so on need to use the detection results as input. In the analysis of agricultural dense scenes, the main challenges of detection are: (1) for the case of quantity-dense scenes, serious occlusion between objects, as well as small objects; (2) for the inner-dense scenes, due to the narrow line spacing, overlapping similar objects often appear. In this context, DL used in detection includes the most advanced object detection networks and semantic image segmentation models.

Weeds detection and control is an important application of precision agriculture [87], and also an important application of dense scenes in agriculture. Weeds generally have the characteristics of wide distribution, high density and low nutritional value, which do not make use of the growth and development of crops. So, it is of great significance to accurately detect weeds in farmland for controlling weeds, spraying pesticides, machine cutting and so on. Dyrmann et al. [69] used DetectNet-based structure consisting of a fully convolution neural network to detect weeds. They used a model trained on the ImageNet to initialize the weight, and by evaluating the error of bounding boxes and predicting the coverage map, the FCN is trained to determine the position of weeds in the image. The network could detect a single weed in a grain field under the condition of serious leaf occlusion. The results showed that the accuracy was 86.6%, although most of the weeds overlapped with wheat plants. The average IOU was 0.64, according to the IOU standard, the algorithm encountered problems in the detection of very small weeds, grasses and weeds exposed to serious overlap, which led to the performance degradation of the system. Due to the selection of the area with the largest occlusion degree, they found a big challenge of automatic weed detection, that is, small target and serious occlusion, which must be solved for the precise control of wheat field weeds. They also pointed out that not all weeds were annotated in the training data used in the study, so if the training data is fully annotated, the accuracy of the test will be further increased. So, some studies have done weed detection on more challenging datasets. Yu et al. [71] reported three deep convolution neural networks (DCNN) including VGGNet, GoogLeNet and DetectNet for detecting weeds in bermudagrass [Cynodon dactylon (L.) Pers.]. The detection results are very accurate, among them, DetectNet is the most successful because it exhibits high performance in multiple images with very high weed density (more than 100 weeds per image), with F1 scores > 0.99. The networks of all of them have all achieved good detection results under severe blocking conditions, and cannot play a role in a few high-density weed detection images.

The precise location of fruits is also an important application in agricultural dense scenes, detecting fruits helps to reduce one of the most labor-intensive tasks in orchards [88]. Different from the challenge of weed detection task, which mainly depends on the deep object detection network to deal with inter-object occlusion, fruit detection in orchard often still faces the problem of occlusion of other objects in the background [89], small objects and different appearance changes. So, in a real outdoor farm environment, fruit detection is more challenging than weed detection, and the DL algorithms based on segmentation network is widely used and dense fruit image detection to separate the object from the background.

Experimental results show that the region-based object detection networks can achieve accurate object detection even in the complex background of light changes. In view of the fact that a single sensor mode cannot fully provide the information needed to detect the target fruit, the fruit detection system based on the multi-modal region has begun to be used in dense agricultural scenes. A high-performance fruit detection system was developed by [33], i.e., a novel multi-modal Faster R-CNN based on VGG-16. By using early and late fusion, combined multiple modes (RGB and NIR) images, they combined the classification decisions of the two modes and changed the structure of the VGGNet input layer. The end-to-end network can significantly reduce the training and prediction time, and further improvements have been made in the detection results. However, the detection results are relatively poor on the fruits with most areas blocked. Bargoti and Underwood [72] proposed a trained model called Tiled Faster R-CNN to implement fruit detection over orchard data containing between 100–1000 fruits per image. The experiment was carried out on the ZF network and VGG-16 net. By using the attention mechanism, the individual proposals are propagated through the fully connected layers and then become the two sibling layers again, and a more refined output of region classification and associated object boundaries were obtained. In addition, in order to perform more advanced tasks on the original image, they used smaller sliding windows to perform detection or "tiling" on the larger image to obtain tiled Fast R-CNN. Recently, a new detection method has been provided by utilizing additional features and machine learning strategies. Dias et al. [73] studied the removal of apple blossoms at a specific growth stage. In view of the fact that the detection of apple flowers is hampered by variable lighting and blocking of leaves, stems or other flowers, they further fine-tuned the Clarifai model [90]. It had been significantly fine-tuned, and effectively combined the features extracted by CNN with color and morphological information, which made their methods have certain applicability in various occlusion level scenes. Even in datasets that are significantly different from training data, the optimal recall rate and accuracy rate are close to 80%. Different from the above two-stage model, Bresilla et al. [31] proposed an end-to-end fast and accurate fruit detection model based on YOLO. By modifying the standard model, the network scale was expanded, some layers were deleted, and then two other blocks are added. The “splitter” block at the entrance divided the image into four separate images, and the “joiner” block at the end splices the four pieces together, taking into account speed and accuracy. However, their models cannot detect two objects of the same category. Accurate fruit detection is also the key to counting based on detection, because any undiscovered fruit will not be counted. Häni et al. [67] compared detection methods using GMM and image segmentation, deep image segmentation network, U-Net and object-based detection network. Among them, their deep object detection network is based on the work of [72], but the backbone network is switched to the ResNet50. In order to make up for the poor performance of the network in detecting small objects, they used a feature pyramid network (FPN) with lateral connections proposed in [49] to extract more low-level features. The counting results also showed that the DL method using 50-layer ResNet is more accurate. Of course, UNET did quite well. Grimm et al. [68] developed a learning semantic segmentation framework for grape detection, localization, and counting. The serial VGG-16 is used as the encoder and the combination of U-Net, SegNet, and FCN-8 is used as the decoder to effectively segment the object and background. Specifically, the decoder used upsampling and downsampling steps with factors of 2 and used concatenations of complete feature maps of the encoder part with feature maps of the decoder part having the same size. Then, the decoder ended with a final upsampling layer with a factor of 8. It can learn more complex models while having good detection performance, and reduce the memory and running time of image segmentation. In addition to the method based on semantic segmentation, case segmentation is also used in agricultural dense object detection. Gonzalez et al. [74] proposed a new instance segmentation algorithm of blueberry quantization based on Mask R-CNN. ResNet101, ResNet50 and MobilNetV1 were selected as backbone networks and several experiments were carried out in each network. Different from the usual method, their algorithm was trained from the beginning on a challenging new dataset captured in the field. In order to detect small objects in dense images, they improved the standard feature extraction pyramid by adding a second pyramid to access lower and higher-level features. Good detection results were obtained in dense images.

### 3.3. Counting and Yield Estimation

Since Seguí et al. [91] began to study the number of interest counted by CNN, the counting method based on DL has become popular. The automatic counting of agricultural products is very helpful for agricultural enterprises to optimize and simplify their harvest. Yield is the criterion for evaluating the production situation and production efficiency, estimation of yield is very important for agricultural producers, and it will be the basis for producers to forecast storage demand, profit and production capacity [92]. In the dense scene of agriculture, counting and yield estimation are similar, because the yield estimation based on DL mainly uses the counting based method. The meaning of counting is to calculate the number of all objects in each frame, while the yield estimation needs to integrate these counts in the whole dataset, and also needs to eliminate duplicate counts. In other words, yield estimation is based on previously obtained data. In general, counting is to estimate part of the yield. It is not hard to imagine that counting and estimating the yield is challenging because it deals with a large number of clustered objects, and the use of artificial methods is time-consuming and laborious, which is not suitable for large-scale cultivation of crops. In dense scenes of the agricultural sector, there are two difficulties: (1) Objects often grow in arbitrarily large clusters. (2) Objects are often occluded by branches, leaves or other objects. Methods of object counting tend to fall within four categories: segmentation-based, detection-based, regression-based, and finally density functions-based.

Segmentation-based methods are used most frequently. French et al. [75] explored a method to segment the scene and counting fish with CNN, which exploited the $N^{4}$-Fields [93] as a foreground segmentation method. The $N^{4}$-Fields generated a real-valued output image giving the probability that each pixel is a foreground pixel. After foreground segmentation, the $N^{4}$-Fields algorithm was used to predict edge maps again, and then small convolution was used to segment and count objects in surveillance video. Chen et al. [9] used a blob detector based on FCN to extract candidate regions, and then used a counting algorithm based on second convolution network to estimate the number of each region. Finally, a linear regression model was used to get the final fruit counting. Since FCN does not use any fully connected layer, the network can perform the partitioning task well, and by initializing weights as the VGG network weights, the network can be trained quickly. Their method can realize good counting results even in very dense conditions. It is very difficult to use only one network to deal with the counting problem of large fruit images which differ greatly in appearance and quantity. Further, Liu et al. [78] combined deep segmentation, frame-to-frame tracking and 3D location technology, proposed a new counting method: using FCN model to segment targets and non-targets, using Hungarian algorithm to track inter-frame results, and finally using 3D location to eliminate the trajectories counted repeatedly and correct the count by rejecting false positives.

YOLO network is the most effective counting method based on the detection. In the research of Zhong et al. [76], they designed and implemented a vision-based flying insect counting system. To detect flying insects, the phenomenon of camera out of focus and impurity interference is easy to occur in the process of image acquisition, so a YOLO network with strong anti-interference ability and reliability is selected. For the convenience of counting, they supposed flying insects as a class and used YOLO for detection and rough counting, which was pre-trained on ImageNet and added a convolutional layer to transform classification model into detection model. Then, the boundary box of detection results of the fine-tuned model is provided to SVM for identification to alleviate the problem of insufficient samples.

Through density regression, we can also train a deep model which can solve the counting task. To solve the problem of crowding and occlusion between targets, Arteta et al. [79] proposed a method to define the target density map by foreground segmentation. Compared with the general density regression, this method is easier to learn, and its core is to use the robustness of fcn8s to noise annotation. Specifically, three tasks, foreground background segmentation, explicit local uncertainty estimation, and density estimation, are solved through a single deep structure joint training. The algorithm performs well in very crowded conditions, but there are still many problems to be solved.

Since all previous fruit counting methods based on DL rely on counting the detected instances. In fact, under the conditions of the large variance of illumination, leaf occlusion or some degree of overlap amongst fruits, in it is difficult to count directly even using DL methods. Rahnemoonfar [77] first proposed fruit counting based on deep simulation learning. In their method, objects were counted without detecting objects. They modified the Inception-ResNet-A layer of Inception-ResNet to capture the characteristics of multiple scales and generated synthetic images for network training, and good counting results were achieved. The experimental results showed that the average test accuracy of their network in the real image and the synthetic image is 91% and 93%, respectively. However, it is still a long way from promoting this method. In their subsequent further research, on this basis, they reduced the image size and the number of expansion filters, realizing a real-time estimation of tomato yield [80].

In addition to the above counting methods, there is also region or area-based method for yield estimation. Based on the fact that the panicle number is directly related to the final yield, yield can be predicted by detecting the leaves and panicles of rice. [81] first used UAV remote sensing data to estimate crop yields using CNNs; they proposed a DL network consisting of two independent branches CNN structures for processing RGB and multispectral images respectively. The first branch consists of five convolutional layers and three max-pooling layers, similar to the reduced version of AlexNet, used to learn spatial features in RGB images. The second branch uses the structure of a pool layer and three convolutional layers to extract features from multispectral images. Due to the difficulty in obtaining high-resolution remote sensing images and the corresponding crop yield distribution, the research on the application of dl to the estimation of production at home and abroad is very limited. It is worth mentioning that Hasan et al. [32] established a wheat spikes dataset, which consists of hundreds of high-quality wheat images with 25,000 annotated peaks, in total. Four region-based convolutional neural networks (R-CNNs) were designed to accurately detect and count spike regions in each map in the study and to better classify and improve the overall detection performance, they made some modifications to the implementation method and optimized the hyper-parameters.

After the objects are detected and counted, to solve the problem that fruit parts are occluded or invisible in the cluttered environment, measures should be taken to improve the robustness of the counting method. Clustering and merging single prediction through cross-frame tracking is a routine operation to avoid repeated counting [77]. Some work combined GPS positioning information to accurately track objects [94,95]. It was later discovered that the accuracy of counting was greatly improved by using multiple perspectives. Das et al. [96] utilized multi-sensor platforms and optical lines, and Hung et al. [97] ensured that the images do not overlap at all by selecting the sampling frequency, thus avoiding tracking. Besides, Structure from Motion (SfM) [98] technology can be used to perform 3D reconstruction, and cluster views can be tracked by estimating camera motion [67]. In short, counting is still a thorny problem in dense agricultural scenes. Generally, the accurate counting results cannot be obtained by using the DL method alone, all of these often need to be combined with other methods to further improve the counting performance.

## 4. Discussion

In the previous section, we reviewed the DL methods and their applications in dense agricultural scenes. In this section, we first present an analysis of the applications of the surveyed DL methods. Then, the limitations and future research directions of DL applications that we think are meaningful in this field are emphasized.

### 4.1. Analysis of the Surveyed DL Applications

(1) Data Preprocessing

**Figure 6:**
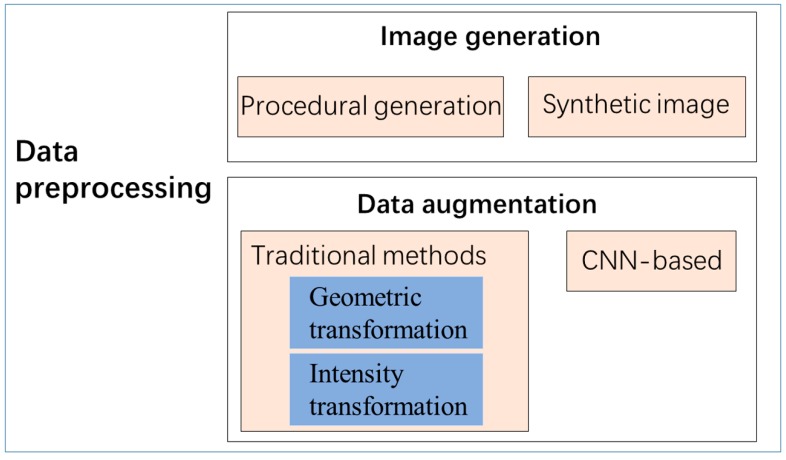
Methods of Data Preprocessing.

One of the major features of DL is the need for a large amount of data. One of the most prominent factors in promoting DL is the emergence of large, high-quality, publicly accessible annotated datasets [99]. The quality and quantity of training data often determine the depth of the network and the effects of the model [24]. To get better training data, many studies have adopted the method of image generation and data augmentation [100]. Figure 6 shows the methods of data preprocessing.

At this stage, most of the datasets studied by DL come from some large technology companies, it is difficult for general researchers to establish high-quality public datasets, but the emergence of data synthesis technology breaks the shackles. Synthesis data is data generated by a computer to simulate real data. There is no cost to generate new data by using synthetic images of computer-generated models to enhance the dataset of images [101], and simply training synthetic datasets can be successfully applied to real data. In addition, it has been proved that the network trained on synthetic data can be generalized into two separate real image datasets, each of which has a different distribution. Generally, the DL model is trained on synthetic data, then validated on actual data, and finally applied to real data. The experimental results showed that the effect of synthetic data training is fine, and only when there is a big deviation between synthetic data and real data, the accuracy will be reduced. Experiments in [101] were implemented using lpfg, a plant simulator based on L-system included in the Virtual Laboratory plant modeling environment. Virtual environments allow free placement of any contained element in a scene and generate its semantic annotations without additional work. In addition to generating synthetic data in a virtual environment, there is a simpler and more widely used synthetic data generation method. Rahnemoonfar [77] filled the entire blank image with green and brown circles to simulate the background and tomato plants and used Gaussian filtering for blurring; then, random circles were drawn at any position to simulate tomatoes with variable sizes. The images overlap with the change of size, proportion, and illumination to fuse complex real scenes.

Data augmentation is commonly used in the use of DL methods to artificially expand training data. There are two purposes of data augmentation: one is that the sample is scarce, which is not enough to support the training of DL networks, so it needs data augmentation to increase the number of the sample. The other one is to prevent over-fitting, over-fitting means that the DL model performs too well in training datasets, resulting in poor performance in testing datasets. Data augmentation usually supplements the original data in quantity and diversity, and we can divide data augmentation methods into traditional methods and DL methods. Traditional augmentation technology can be divided into geometric transformation and intensity transformation. The geometric transformation includes size adjustment, sample tailoring, rotation, inversion and so on. The intensity transformation includes the transformation of contrast, brightness, color, and noise. Besides, mixup and PCA enhancement technologies are also traditional data augmentation means. Recently, methods based on DL for data augmentation have become popular. The CycleGAN [102] is essentially two mirror-symmetrical GAN, it can learn the characteristics of a class of data and generate similar data. The research showed that compared with traditional image enhancement methods, the CycleGAN method greatly enriched the diversity of training data sets [103].

Although data augmentation technology and synthetic image technology expand some data sets, greatly alleviating the problem of lack of data sets, in fact, at least hundreds of images are needed, which depends on the complexity of the problem studied.

(2) Surveyed models and model optimization

Although DL has made substantial progress in many computer vision problems, up to now, the research on dense scenes in agriculture is not mature. However, the review shows that the DL methods are better than the traditional machine learning methods based on the performance index adopted by the authors. Our analysis shows that in most of the related work, the main DL method to deal with dense scenes in agriculture is CNN, among which VGGNet and ResNet are the most commonly used models. Since the closer the network is to the bottom, the more effective the extracted features are, it can be said that the network architecture of VGGNet is superior to other most advanced networks. So, in order to maintain this characteristic, many convolutional neural network models use VGGNet as the basic model for image detection and other tasks. ResNet can not only prevent the degradation of deep neural network, but also improve the training quality of the model, so it is widely used in dense scenes analysis. As reviewed above, data annotation in dense images is very difficult. As a result, due to the character that it needs less data and can use available data accurately, semantic segmentation network including FCN and U-Net is also widely used to solve recognition, detection and counting tasks by segmenting foreground objects.

CNNs are the most important deep learning methods in the dense agricultural scene. At the same time, transfer learning and autoencoder are also used in the dense agricultural scene. The biggest use of transfer learning is to alleviate the lack of data and pre-training the networks. Supervised learning needs a lot of annotation data, which is also one of difficulties of DL in dense scenes. On the one hand, many machine learning methods work only when training data and test data come from the same feature space and have the same distribution. If the distribution of data changes, most models need to be reconstructed [104]. On the other hand, labeling data is a tedious and expensive task. Reducing the amount of data means reducing the overhead, but there is a risk of reducing network performance. Therefore, due to the low data requirements [105], transfer learning is more and more used in the applications of DL. Transfer learning allows different domains, tasks, and distributions of training and testing data while saving a lot of labeling work. It is also helpful in adapting a training model for one period of time or equipment so that it can be applied to another period of time and transferring knowledge to a new field [106,107,108]. In computer vision tasks, it has become a standard operation to transfer the trained features (i.e., fine-tuning) from the trained CNN to the new target tasks [72]. In general, the pre-training model and initial weights are obtained by pre-training on the ImageNet dataset or Pascal VOC dataset. That is to fine-tune the network. Fine-tuning usually requires only a small number of parameters to optimize, and the whole network has not been trained from the beginning. This greatly reduces data requirements for specific tasks. Initialization of convolutional neural networks using models trained on different datasets will result in faster convergence of the networks. After training on thousands or even more annotated images, the DL networks can be better trained in dense agricultural scenes. An autoencoder can also be used to generate data, but it has not yet been used in dense agriculture scenes.

Standard DL models cannot perform dense scenes analysis well, so it is necessary to enhance the basic network architecture. The review shows that there are many methods to optimize the structure of DL network and make it more suitable for dense scenes analysis. One is to integrate different CNNs networks and combine the advantages of each network to deal with occlusion, complex background, and other issues. For example, Grimm et al. [68] designed a segmentation network that combines U-net, SegNet and FCN-8 to segment objects more effectively. The other is to modify the advanced networks, such as changing the size of the convolutional filter, adding or deleting layers of network, updating or adjusting model parameters, etc. [73]. At the same time, there is another network improvement method that has a good performance in the dense agricultural scenes analysis, i.e., CNNs models combined with other classical machine learning methods. Bozek et al. [65] combined U-Net and recurrent components. Zhong et al. [76] used YOLO to count roughly, and then used SVM to classify and recognize accurately. There is also work to divide the objects into clusters, and then detect or count each cluster [67].

There are many commonly used evaluation indexes for DL. Our review shows that accuracy, recall, and IOU are the most frequently used. Among them, the accuracy rate is best understood, which is an index presenting the proportion of correctly classified samples to the total number of samples. Generally, the accuracy rate is used to evaluate the global accuracy of the model, but it cannot fully evaluate the performance of a model. A similar indicator is the recall, which is the proportion of all positive samples in the test set that are correctly identified as positive samples. IOU is an object detection task evaluation index, which is the percentage of overlapping area between detected and ground truth boundary box in the joint area.

(3) Hyperparameters and optimization technology

Hyperparameters are user-defined and tailored to a specific application task. Hyperparameters have a great influence on DL networks, and the performance of networks is likely to depend on subtle differences of hyperparameters. The setting of hyperparameters is an important work in DL, learning rate, batch size, momentum and weight decay are the most common hyperparameters. The deployment of this work often depends on experience. From the relative literature review, the learning rate is discussed more in the hyperparameter optimization technology, and the setting and optimization of other hyperparameters are less involved. Specifically, there are four ways to set the learning rate: (1) fixed learning rate; (2) linear decay policy, when the number of iterations reaches the upper limit, the training stops; (3) exponential decay; (4) step strategy, we set an iteration number in advance, when the number is reached, the learning rate will be reduced to a certain value. Gradient descent is a common optimization technique in machine learning algorithm. Batch gradient descent and stochastic gradient descent (SGD) are used most in the application of dense agricultural scenes. In addition, Ada-Delta and mini batch gradient descent are also applied. Among them, mini batch gradient descent adds extra hyperparameter to the learning algorithm, the batch size. The batch size and weight decay remain unchanged during training. In the literature we investigated, the momentum is generally the same, and the momentum is generally set to 0.9. In order to make the deep learning model provide the best results, it is necessary and not easy to find the best value of these hyperparameters. Therefore, better hyperparameters optimization technologies need to be proposed.

(4) Solutions to challenges

As mentioned in this review, occlusion is the biggest challenge in dense scenes. There are two main methods to alleviate the problem of occlusion. One is to label the object into different categories according to the occlusion situation, and then recognize and classify it [64]. This method avoids the huge error caused by the same feature extraction for all objects. The other is to train DL networks on thousands of large annotated data sets with different sizes, illumination, and occlusion levels, which is the main solution [69]. However it also means collecting large annotated datasets. In the experiment of [79], it took more than three years to collect Penguin datasets and more than 35000 volunteers to label the images. 

Small object is also one of the key challenges in dense scenes. Such a challenge is relieved by optimizing the network. Because the pooling layer in CNNs reduces the feature mapping by two times every time, it does a poor job in detecting small objects. Because the FPN with lateral connections makes up for the difference of spatial resolution in the higher level, it is very suitable for small target detection. In addition, for networks with anchor scale or mini-mask shape, the settings of these two parameters can be changed to improve the detection effect of small objects.

For the case of high background noise, one solution is to select a high-performance network; the other is to add a variety of scenes with high background noise in the test dataset. Some CNNs used for saliency detection are fine-tuned [73] and others choose networks with strong anti-interference ability and reliability, such as YOLO [76], R-CNN, etc. Although DL has made great progress in dense image processing, there is still room for further improvement in serious occlusion, real-time detection and other aspects. Only relying on the network often cannot perform well in the agricultural complex real scene. It is also necessary to train and test representative visual changes and various high noise datasets to improve the robustness of the network.

Besides, in dense agricultural scenes, for background processing with high similarity to the object, using semantic segmentation to extract the region of interest can achieve good results [66,78].

### 4.2. Limitations to DL Applications

(1) Influential Architectures

In general, by considering partially occluded objects in training data or detecting partially occluded objects through design models can improve performance. However, by selecting the densest area, it is possible to reduce the performance of the model. It has been proved that the DL methods cannot be extended well when there are clutter and occlusion in the image [109]. The performance of the network will decrease with the increase of the complexity of the dataset. Moreover, in the dense scenes of agriculture, there are objects that are hard to see even with human eyes. Therefore, the DL algorithms cannot solve the problem of serious overlap up to now.

(2) Datasets

In addition to the fact that DL algorithms are still difficult to deal with serious occlusion problems, the collection, and labeling of dense objects datasets is a major problem. The costs of generating new data are high, it takes a lot of time and effort to acquire and annotate datasets. We are familiar with common datasets for DL architecture, but in the field of agricultural applications, there are few public datasets available. In the complex background of dense agricultural scenes, the construction of datasets is even more difficult. Besides, the datasets used by one research institute are often not suitable for other studies. From Table 2, we can see that all the experiments except one study that used other people’s datasets were self-generated datasets. At present, there are no public datasets for dense agriculture scenes, and few studies can download data directly from the Internet [110], and most require researchers to build their datasets. For example, to collect images of pests and diseases of tomato plants according to the time, the researchers collected 9230 pictures of wheat crops with different wheat diseases under various conditions [111].

### 4.3. Future Work

(1) DL optimization

Facing the challenges of dense scenes analysis in agriculture, given the current solution to the use of auxiliary means to solve occlusion, scale inconsistency, and other issues, the effect is unlikely to have a qualitative leap, the research focus should also turn to the DL algorithm itself. First, we need to add more layers to improve the performance of the DL network. Plain networks do not perform well in dense scenes, so it is necessary to build a deeper DL structure to deal with more complex problems. However, blindly pursuing the layers of the network may lead to too many model parameters, increased computational complexity, and low training efficiency. Therefore, on the premise of not reducing the training efficiency of the network, increasing the number of network layers will be an important research direction to improve the ability of DL in dense scenes analysis. Therefore, it is a trend to explore lightweight CNNs, which can obtain higher recognition accuracy with less trainable parameters [112,113], such as MobileNet. It is possible to propose some new network structures to deal with dense objects. The new compression layer and optimization technology should be considered in the new DL architecture. Next, it is recommended that an in-depth study of the mechanism of DL. In the existing DL applications, the design and optimization of the algorithm mainly focus on the accuracy of the algorithm, rather than studying the black box. Needless to say, DL has a powerful ability, but the process is too abstract. We do not know how it works at present. As a result, Geoffrey Hinton, the father of neural network, said that DL entered a bottleneck period. Therefore, further research on DL theory needs more. Multi-feature fusion is also an effective way to improve DL performance. One is the fusion of multiple features, for example, the combination of spatial features and semantic features can maintain the positioning accuracy and more semantic information [114]. Second, multi-layer features fusion can effectively use spatial information by giving different weights to different feature layers [115]. The third is to combine CNN features with artificial features [116]. In fact, this has been applied in agricultural intensive scenes, but there are more feature combinations, which may achieve the effect of improving network performance. In addition, it is suggested that CNNs combine with other DL algorithms, such as RNN, LSTM. Each DL method has its own application areas. Other DL methods may not be as powerful as CNNs in scenes processing, but they can use their advantages to complement CNNs. Now there are examples of CNNs and RNNs combination [117] and CNNs and LSTM combination [118]. The next step is to improve them to effectively apply them to dense scenes in agriculture. Besides, how to ensure the accuracy of deep learning in agricultural tasks is also a problem to be considered. Although there may be errors inevitably, it is worth studying how to reduce the errors by using the real values for calibration.

(2) To meet the challenges of big data processing

As mentioned in the review, there is no public dense image data set for agriculture. To maximize the potential of DL, it is very important to create a well-annotated large-scale intensive agricultural image database. It is also helpful for researchers to use the same data set for training, verification, and testing, and algorithm comparison. However, it is difficult due to that labeling data is costly and laborious.

Several methods can be used to alleviate the difficulty of datasets generation. One is to generate virtual training data by simulation. There are two issues to consider when applying data generation in an agricultural scene. One is that the generated image is highly similar to the real image, ensuring that it can be tested on the real data set; the other is that the generated image is rich in type, covering all real scenes. Its advantage is that it can express multi-level details and has high modeling efficiency, i.e., it can generate a large amount of data in a very simple way. However, it ignores the complex background in reality, so the difficulty now is to extend the model of synthetic data training to actual data. Therefore, it is a promising method to use the computer to generate a large number of available synthetic data and use these weakly annotated training data to promote the solution of the visual task [119]. Another one is to transform supervised DL into unsupervised or semi-supervised DL. At present, in the field of DL, the use of annotated data for supervised learning is in a leading position. However, with the increase of dense scenes, it is unrealistic to mark all objects. Therefore, there is a lot of research space for semi-supervised and unsupervised DL methods using only part of annotated data. In addition, more and more attention will be paid to the research of automatic annotation. On the other hand, low resolution is another obstacle to the application of DL in dense agricultural scenes, so it is necessary to make a new attempt on low-resolution data based on the DL model.

(3) Apply DL to agriculture through transfer learning

DL is applied to agriculture after achieving excellent performance in other fields. So the application level of DL in other dense scenes is far higher than that in agriculture. For example, in the crowd analysis, many improved DL algorithms or DL combined with traditional machine learning methods are proposed. A lot of open datasets have been generated. Based on transfer learning, transferring knowledge from one application field to another is a learning method that is conducive to the application of DL in agriculture. In the long run, this is a very promising application.

(4) Robot system

The research on robots for weeding, feeding, pruning, picking, and harvesting is a hotspot in agricultural application, which greatly improves labor efficiency, saves production costs, and promotes the development of agricultural machinery. Agricultural robots have developed into one of the most promising industries in precision agriculture [120]. Developing an accurate, real-time and reliable computer system for pest identification or yield estimate is an important part of agricultural robot system. It requires complex algorithms to overcome realistic challenges, such as lighting, shape, occlusion and natural changes in viewpoints [121]. The positioning algorithms and modules corresponding to the robot system are also necessary. Combining robot path planning, mechanical design, and DL algorithm to solve practical needs is also the next research focus. As a visual task, tracking is more and more needed in the dense scene analysis of agriculture. After the object in the dense scene is detected, the research of quantitative trajectory reconstruction algorithms will be an important direction in the future. At the same time, it is necessary to combine DL algorithms with modern technologies such as meteorological information and geographic information to form a comprehensive service platform. By considering the portability of mobile platforms such as Android or ios [122], it can be applied in smart mobile terminals to provide higher practical and promotional value [63].

## 5. Conclusions

As discussed in this review, the greatest challenge for dense scenes analysis is that objects are too dense and small. This paper performed a review of DL in dense agricultural scenes, mostly from an application perspective. In this review, we review the recent research on DL for application in dense agricultural scenes. Based on these surveys, readers can understand which dense scenes exist in current agricultural applications, and which DL algorithms are used in these dense scenes, as well as the application of DL in general agricultural tasks. By comparing the DL methods with other methods in the survey paper, it shows that DL provides better performance in dense scenes and is superior to other popular image processing techniques. In short, research on the application of DL in dense agricultural scenes is a very meaningful and urgent matter. However, what we also need to know is that research on dense agricultural scenes is still in its infancy and many obstacles need to be overcome, such as datasets. The future development of DL technology still has different opportunities and challenges and has a high development prospect.

## Figures and Tables

**Table 1:** Characteristics of various backbone networks in dense images.

Networks	Number of Layers	Architectures	Highlights in Dense Images	First Used in
VGGNet	11/13/16/19	Conv + FC	Very small convolutional filter size and the increase of network depth effectively improves network performance	[36]
GoogLeNet	22	Conv + Inception + DepthConcat + FC	Multiscale convolution and reorganization extract features of different scales	[13]
ResNet	18/34/50/101/152	Conv + ReLU + Residual block + Shortcut + FC	ResNet architecture is deeper through residual block and easier to optimize	[37]
DetectNet	-	-	Predicting the existence of the object and the position of the object relative to the center of the grid square	-
DarkNet	19/53	Conv + Residual layer	Recognizing background correctly and detecting most occluded and overlapped objects	-
FCN	8/16/32	Conv	Accepts input images of any size and is more efficient	[38]
SegNet	13	Conv + up-Conv	Improve the edge depiction and reduce the training times	[39]
U-Net	9	Conv + up-Conv	Each pixel can be segmented, with higher segmentation accuracy and support a small number of training models	[40]

Number of layers: Total number of convolution layers and fully connected layers, Conv: Convolution layer, FC: Fully connected layer.

**Table 2:** Applications of deep learning networks in dense images in different agricultural tasks.

Application	Model	Description of Method	Accuracy (%)	Computing Time	Ref.
Recognition	ResNet	Using 50-layer and 101-layer depth residual networks	50-Layer:94.6/101-Layer:101:98.67	x	[63]
Recognition	YOLOv3/ResNet 152/ResNet50	Yolov3 and two Mark R-CNNs with ResNet-50 and ResNet-152	MaskRCNN-152: 86.83	√	[64]
Recognition	U-Net	Combining U-Net and a recurrent component	-	-	[65]
Classification	U-Net + VGG-16	VGG-U-Net initialized by VGG-16 for segmentation, and VGG-16 for classification	90	-	[66]
Detection	GMM/U-NET	The detection methods of using GMM and image segmentation, U-NET and object-based detection network are compared	91.27	√	[67]
Detection	FCN	An encoder and a decoder part based on the idea FCN	-	-	[68]
Detection	DetectNet	Consisting of a FCN based on the GoogLeNet architecture and clustering function	86.6	√	[69]
Detection	Caffe reference + VGGNet	DeepAnomaly algorithm that using DL combined with anomaly detection	-	√	[70]
Detection	VGGNet/GoogLeNet/DetectNet	Using VGGNet, GoogLeNet and DetectNet for detecting weeds in bermudagrass	-	-	[71]
Detection	ZF network and VGG-16	Faster R-CNN with ZF network and VGG-16, and different types of transfer learning are used to initialize the network	95.8	√	[72]
Detection	YOLO	Modified YOLO with two other blocks. The “splitter” block at the entrance divided the image into four separate images, and the “joiner” block at the end splices the four pieces together	87	√	[31]
Detection	VGG-16	VGG-16 with early and late fusion, and combining multiple modes (RGB and NIR) images	-	√	[33]
Detection	Author-defined CNN	Fine-tuned the Clarifai model combined CNN with color and morphological information	92.0	-	[73]
Detection	ResNet101/ResNet50/MobilNetV1	An instance segmentation algorithm based on mask T-CNN. Experiments on ResNet101, ResNet50 and MobilNetV1	-	√	[74]
Counting	Author-defined CNN	$N^{4}$-Fields algorithm is used to foreground segmentation predict edge maps	90.39	√	[75]
Counting	FCN	A blob detector based on a FCN	-	√	[9]
Counting	YOLO+SVM	Adding convolutional layer to the pre-trained YOLO model transforms classification model into detection model	93.71	√	[76]
Counting	Inception-ResNet	Modified the Inception-ResNet-A layer to capture the characteristics of multiple scales	91	√	[77]
Counting	FCN	Using FCN model to segment targets and non-targets	-	√	[78]
Counting	FCN-8	Enhance and interleave density estimation by foreground background segmentation and explicit local uncertainty estimation			[79]
Yield Estimation	Inception-ResNet	Modified the Inception-ResNet-A layer to capture the characteristics of multiple scales	91	√	[80]
Yield Estimation	AlexNet	Two independent branches CNN structures for processing RGB and multispectral images respectively	-	√	[81]
Yield Estimation	R-CNNs	R-CNNs optimized for implementation method and hyper-parameter	93.3	√	[32]

**Table 3:** Datasets in agricultural dense scenes.

Ref.	Description of Datasets	Type of Image (Is It a Real Image)	Resolution(Pixels)	Objects and Numbers for Training		
				Objects	Training	Validation/Testing
[63]	Dataset obtained from [82]	√		Pest	800	300
[64]	300 images captured by binocular color camera in Fruit Garden and Citrus Experimental Base	√	1024 × 768	Fruit	250	50
[65]	A dataset of 8, 640 30 FPS and 12, 960 70 FPS images containing total of 375, 698 annotated bees	√	512 × 512	Bees	19,434	2176
[66]	500 photos in the sunflower field taken by robot	√	512 × 384	sunflower	1500	350/150
[67]	Datasets were acquired by mobile phones in HRC over a period of 2 years.	√	500 × 500	Apples	5200	1300
[68]	Six sets of grape image data provided by Grape Breeding Institute	√	5472 × 3648	ShootsN	35	24/36
				ShootsA	34	34/34
				InfloresN	15	15/15
				PedicelsA	40	40/40
				GrapesN	30	30/30
				GrapesA	30	30/30
[69]	A dataset consisting of 1,427 images from 10 fields	√	1224 × 1024	Weeds	1274	153
[70]	104 images on the grass by a stereo camera	√	1080 × 1920	Field	56	48
[71]	Datasets were taken at multiple athletic fields, golf courses, and institutional lawns in FL.	√	640 × 360	Hydrocotyle spp.	12,000	1000
				Hedyotis cormybosa	16,132	1000
				Hedyotis cormybosa	13,206	1000
				Poa annua	12,030	1000
				Multiple-species	36,000	1000
[72]	Capture image data by sensors in different orchards	√	500 × 500	Apples	729	112
				Almonds	1154	270
				Mangoes	385	100
[31]	In total 100 images of apples and 50 of pears were taken.	√	1216 × 1216	-		
[33]	Some data sets are collected by themselves, while the rest are from Google’s image search.	√	1296 × 964 and 1920 × 1080,	Sweet pepper	100	22
[73]	Datasets of 147 images from the apple tree were collected by camera	√	5184 × 3456	Apple	100	47
[74]	A dataset of 293 images taken in the field and an annotated dataset of 10,161 blueberries for detection and 228 for segmentation.	√	images with 3264 × 2448/videos with 1920 × 1080	Blueberry-V1	-	-
				Blueberry-V2	-	-
[75]	A dataset contains 52 videos from six different conveyors with a total of 443 frames.	√	-	-		
[9]	Orange datasets without lighting during the day and Apples data at night	√	1280 × 960	Oranges	3600	35
				Apples	1100	10
[76]	Collection of dataset using capture devices and image	√	30 × 30	-		
[77]	26400 synthetic images produced by the authors	Synthetic images	-	Synthetic tomato	24,000	2400
[78]	Orange datasets without lighting during the day and Apples data at night	√	-	-		
[79]	More than half a million images have been generated in more than 40 different locations over three years	√	-	Penguin	57,400	24,600
[80]	26,400 synthetic images produced by the authors	Synthetic images	-	Synthetic tomato	24,000	2400
[81]	There were 643 areas of interest in total	√	1280 × 960	Paddy rice	412	102 for validation and 129 for test
[32]	335 pictures of 10 wheat varieties at three different growth stages	√	5184 × 3456	Wheat	305	30
